# Therapeutic Potential of Erythrina Genus: Bioactive Phytoconstituents with Potent Antiviral and Antimicrobial Activities

**DOI:** 10.3390/plants14193053

**Published:** 2025-10-02

**Authors:** Muchtaridi Muchtaridi, Samuel Lestyawan, Maitsa Alya Fakhirah, Agus Rusdin, Shela Salsabila, Sandra Megantara, Anas Subarnas, Nur Kusaira Khairul Ikram

**Affiliations:** 1 Department of Pharmaceutical Analysis and Medicinal Chemistry, Faculty of Pharmacy, Universitas Padjadjaran, Sumedang 45363, West Java, Indonesia; agus18002@mail.unpad.ac.id (A.R.); shela24001@mail.unpad.ac.id (S.S.); s.megantara@unpad.ac.id (S.M.); 2 Research Collaboration Center for Radiopharmaceuticals Theranostic, Universitas Padjadjaran, Sumedang 45363, West Java, Indonesia; 3 Apothecary Program, Faculty of Pharmacy, Universitas Padjadjaran, Sumedang 45363, West Java, Indonesia; samuel21001@mail.unpad.ac.id (S.L.); maitsa21001@mail.unpad.ac.id (M.A.F.); 4 Department of Pharmacology and Clinical Pharmacy, Faculty of Pharmacy, Universitas Padjadjaran, Sumedang 45363, West Java, Indonesia; a.subarnas@unpad.ac.id; 5 Institute of Biological Sciences, Faculty of Science, Universiti Malaya, Kuala Lumpur 50603, Malaysia; nkusaira@um.edu.my

**Keywords:** erythrina, antiviral activity, antimicrobial resistance, phytoconstituents, flavonoids, infectious diseases

## Abstract

Infectious diseases present a significant global health challenge, further exacerbated by the rising prevalence of antimicrobial resistance and the limited availability of effective antiviral and antimicrobial agents. The Erythrina genus has garnered scientific interest due to its diverse array of bioactive phytoconstituents, with potential therapeutic relevance. This review aims to synthesize and critically assess the existing literature on the antiviral, antibacterial, antifungal, and antiplasmodial properties of Erythrina species. A comprehensive literature search was conducted using PubMed, Scopus, and Google Scholar databases. Relevant studies were identified through keyword searches combining pathogen-specific terms with “Erythrina”. The extracted data were categorized based on the pathogen type and its associated bioactive compounds. Several Erythrina species exhibited substantial antiviral activity against prominent viral pathogens, such as HIV and SARS-CoV-2. Notably, strong antibacterial efficacy was observed against *Staphylococcus aureus*, including multidrug-resistant strains. Antifungal activity was most pronounced against *Candida albicans*, while potent antiplasmodial effects were reported against both drug-sensitive and drug-resistant strains of *Plasmodium falciparum*. These pharmacological effects were predominantly attributed to prenylated flavonoids, isoflavones, pterocarpans, and erythrina-type alkaloids. Further mechanistic studies and in vivo evaluations are essential to fully assess their clinical efficacy and support the development of plant-derived antimicrobial agents.

## 1. Introduction

Infectious diseases are caused by pathogens such as bacteria, viruses, fungi, and parasites, and continue to pose a major global health concern [1]. According to the World Health Organization (WHO), infectious diseases remain one of the leading causes of morbidity and mortality globally, exacerbated by the rise in antibiotic-resistant strains and the emergence of novel pathogens [2]. Current treatment modalities, including antibiotics and antiviral drugs, are becoming less effective due to pathogens’ resistance mechanisms and the limited development of new drugs [3]. Consequently, there is a growing interest in exploring alternative therapeutic sources, such as plant-derived compounds, which have historically been a rich source of novel bioactive agents with potential therapeutic benefits [4].

Erythrina plants, commonly known as “coral trees”, belong to the Fabaceae family and are renowned for their diverse medicinal properties. These plants have been traditionally utilized in diverse cultures for their purported antiviral and antimicrobial properties. Recent studies have identified several bioactive compounds from the Erythrina genus that demonstrate promising antiviral and antimicrobial activities [5,6]. Such findings suggest that Erythrina species could offer valuable alternatives or supplements to conventional treatments, addressing the pressing need for novel therapeutic agents against resistant pathogens and emerging infectious diseases.

This review aims to synthesize and critically evaluate the existing research on the antiviral and antimicrobial properties of Erythrina species. By summarizing identified bioactive compounds and their mechanisms of action, this review underscores the genus’s potential as a source of novel therapeutic agents. This synthesis of existing knowledge serves as a guide for future research directions and encourages further investigation into the clinical applications of Erythrina-derived compounds in the management of infectious diseases.

The persistent threat of infectious diseases continues to pose a significant global health challenge, resulting in substantial morbidity and mortality across both developing and industrialized nations. Despite substantial advancements in modern medicine, the emergence of antibiotic-resistant bacterial strains and novel viral pathogens, such as SARS-CoV-2, has undermined the effectiveness of current therapies [2,3]. The declining pipeline of novel antimicrobial agents further intensifies this crisis, underscoring the need to explore alternative and supplementary strategies to conventional pharmacotherapy.

The current frontline approach relies heavily on synthetic antimicrobials, including antibiotics, antifungals, antiprotozoals, and antiviral agents. While these therapies have improved global health outcomes, their long-term efficacy is limited by adaptive resistance mechanisms, narrow pathogen specificity, and adverse safety profiles. Resistance to β-lactam antibiotics, neuraminidase inhibitors, and even artemisinin-based compounds has been reported across diverse clinically relevant pathogens [4]. This has prompted the scientific community to reexamine the therapeutic value of natural products, particularly those derived from medicinal plants, which often exhibit multi-target activities and lower risk of resistance development.

Among the diverse botanical sources with ethnopharmacological relevance, Erythrina species are particularly notable for their longstanding ethnomedicinal use in Africa, Asia, and the Americas. These plants produce a wide range of secondary metabolites, including prenylated flavonoids, erythrina-type alkaloids, and pterocarpans, many of which exhibit potent antimicrobial, antiviral, antifungal, and antiplasmodial activities [6,7]. Several Erythrina species have demonstrated efficacy against priority pathogens such as *S. aureus*, *C. albicans*, *Plasmodium falciparum*, and even HIV and SARS-CoV-2, thereby positioning the genus as a promising reservoir of bioactive compounds for future drug development [8,9].

**Figure 1:**
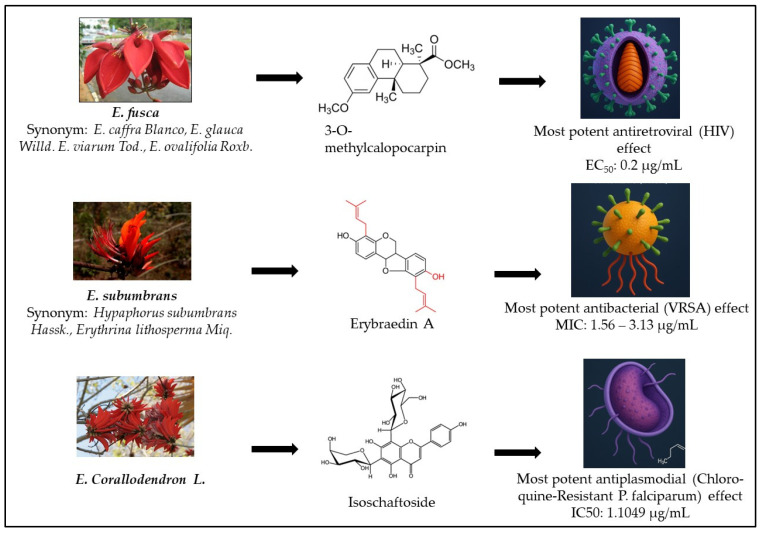
Review for the most potent Erythrina sp. as an antivirus, antibacterial, antifungal, and antiplasmodial agent.

Despite numerous studies, with 81 published investigations collected from Scopus, PubMed, and Google Scholar databases reporting on the biological activities of isolated compounds and crude extracts from Erythrina species, including [10,11,12,13,14,15,16,17,18,19,20,21,22], antibacterial [23,24,25,26,27,28,29,30,31,32,33,34,35,36,37,38,39,40,41], antifungal [42,43,44,45,46,47], and antiplasmodial [48,49,50,51,52,53,54,55,56,57,58,59] properties, systematic and mechanistically oriented reviews remain limited. Existing reviews primarily concentrate on ethnobotany or individual pharmacological aspects, frequently disregarding integrative evaluations such as compound–pathogen interactions, comparative species analyses, and the potential role of Erythrina-derived compounds in combating antimicrobial resistance. Additionally, correlations between extract composition and in vitro potency, as well as cross-pathogen activity trends, have not been comprehensively examined. This review aims to address these gaps by providing a critical and integrative synthesis of the antiviral and antimicrobial properties of Erythrina species. By discussing phytoconstituent profiles, reported bioactivities, and proposed mechanisms of action, this review highlights the genus’s pharmacological importance and therapeutic potential. Ultimately, it seeks to inform and guide future translational research, supporting the advancement of Erythrina-derived compounds as promising candidates in combating infectious diseases and multidrug-resistant pathogens, as illustrated in Figure 1.

Figure 1 shows the broad spectrum of activities exhibited by Erythrina species, including antivirus, antibacterial, antifungal, and antiplasmodial effects. The most potent antiviral activity was observed in *E. glauca* against HIV, attributed to the active compound 3-O-Methylcalpocarpin, and in *E. sigmoidea* against SARS-CoV-2, linked to the compound gangetin. The most potent antibacterial activity was demonstrated by the methanol extract of *E. caffra* stem bark against *S. aureus*. In terms of antifungal activity, the ethanol stem bark extract of *E. senegalensis* showed the greatest potency against *C. albicans*. For antiplasmodial activity, the methanol root bark extract of *E. sacleuxii* exhibited the most significant effect against *P. Falciparum*.

## 2. Results

### 2.1. Antiviral Properties of Erythrina

*Erythrina glauca* has been reported to exhibit anti-HIV activity. Two compounds isolated from this species, sandwicensin and 3-O-methylcalopocarpin, demonstrated EC_50_ values of 2 µg/mL and 0.2 µg/mL, respectively [11]. These values indicate greater potency compared to compounds from *Erythrina lysistemon*, specifically 5-deoxyglyasperin F and 2′-hydroxyneobavaisoflavanone. These compounds exhibited significantly higher EC_50_ values, ranging from 11.5 µg/mL for 5-deoxyglyasperin F and 7.6 µg/mL for 2′-hydroxyneobavaisoflavanone [12]. A crude alkaloid fraction isolated from *Erythrina abyssinica* exhibited an EC_50_ value of 53 µg/mL, demonstrating lower potency compared to both *E. glauca* and *E. lysistemon*, despite the presence of bioactive compounds such as erythraline, erysodine, and related alkaloids [17]. In contrast, apigetrin from *Erythrina variegata* exhibited an EC_50_ of 100.59 µg/mL, demonstrating the least potential anti-HIV effect [10]. Notably, *Erythrina senegalensis* yielded diverse anti-HIV prenylated flavonoids, with 6.8-diprenylgenistein (EC_50_: 0.5 µM) exhibiting exceptional potency, surpassing all other compounds reported [12].

Phukhatmuen et al. [14] reported anti-SARS-CoV-2 activity in *Erythrina subumbrans*, isolating gangetinin as the active component, although specific EC_50_ values were not reported. Similarly, Nkengfack et al. [13] identified gangetin in *Erythrina sigmoidea* with comparable antiviral claims against SARS-CoV-2. Togola et al. [16] reported erybraedin D from *Erythrina senegalensis*, while Tanaka et al. [15] and Desta et al. [18], identified orientanol E from *Erythrina variegata* and erycaffra F from *Erythrina caffra*, respectively. However, in most cases, potency data (EC_50_ values) were not reported, limiting direct comparison.

Beyond HIV and SARS-CoV-2. other viruses have been investigated. Fahmy et al. [19] explored the anti-hepatitis viral activity of vitexin from *Erythrina speciosa*, which exhibited an EC_50_ of 125 µg/mL. In contrast, Hubert et al. [20] found significantly higher potency in compounds isolated from *Erythrina senegalensis*, such as 2.3-dihydro-2′-hydroxyosajin, osajin, and 6.8-diprenylgenistein, all with EC_50_ values around 67–72 µg/mL. These results indicate that *E. senegalensis* exhibits stronger anti-hepatitis viral activity than *Erythrina speciosa*. Another study analyzed *Erythrina addisonae* for anti-influenza potential and reported EC_50_ values ranging from 8.8 µg/mL to 26.44 µg/mL across several prenylated flavonoids, indicating considerably higher antiviral potency relative to both *Erythrina speciosa* and *E. senegalensis* [8].

Mollel et al. [21] investigated the anti-herpes simplex virus (HSV) activity of *Erythrina abyssinica*, noting an IC_50_ of 27 µg/mL from its stem bark aqueous extract. In a comparative context, Fahmy et al. [6] reported that vitexin from *Erythrina speciosa* showed an EC_50_ of 64 µg/mL, suggesting moderate activity. Pino et al. [60] evaluated *Erythrina fusca* and found its stem bark decoction had an EC_50_ of 243 ± 10.9 µg/mL, representing weaker activity among HSV-related studies. Similarly, González-Lavaut et al. [22] observed *Erythrina poeppigiana* to be slightly more potent than *E. fusca*, with an EC_50_ of 147.6 ± 4.3 µg/mL, yet still less active than *E. abyssinica* or *Erythrina speciosa*.

Finally, Rasool, et al. [61] reported the antiviral activity of *Erythrina variegata* against the Dengue virus, identifying multiple bioactive flavonoids including ericristagallin, osajin, and sigmoidins A–C. While no EC_50_ values were reported, the presence of these structurally diverse compounds aligns with the genus-wide trend of broad-spectrum antiviral potential observed in earlier studies. Table 1, presented below, provides a concise summary of several Erythrina species that have been tested for their antiviral activity.

*The Erythrina* genus is a potential inhibitor for the SARS-CoV-2 RNA-dependent RNA polymerase (RdRp), a critical enzyme for viral replication. Herlina et al. (2025) screened out 473 flavonoids from the genus *Erythrina* against the viral RNA-dependent RNA polymerase (RdRp) enzyme of the SARS-CoV-2 virus using computer-aided drug design [63]. The *Erythrina* genus, particularly butein, presents highly promising candidates for the development of SARS-CoV-2 RdRp inhibitors. Butein’s robust binding affinity, distinctive inhibitory mechanism, and favorable predicted safety profile establish a strong foundation for future experimental (in vitro) studies to substantiate its antiviral efficacy against COVID-19.

**Figure 2:**
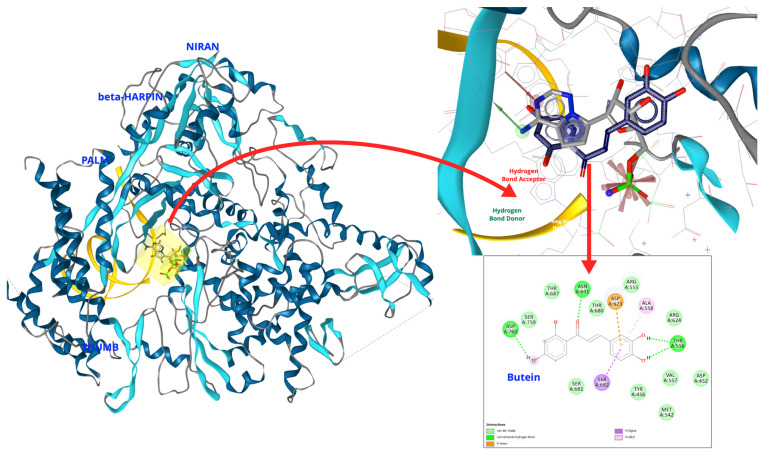
Molecular docking simulation of butein (blue carbon) and remdesivir (gray carbon) by LigandScout 4.2 (under license acquired from Universitas Padjadjaran). Magenta highlights: negative ionizable; green highlights: hydrogen bond donor; red highlights: hydrogen bond acceptor; and blue highlights: ring aromatic hydrophobicity.

Similar to remdesivir, butein interacts with specific residues and nucleotides of the RNA primer and template of the SARS-CoV-2 virus. Figure 2 illustrates that butein is located between the primer and template RNA when interacting with amino acid residues in the palm subdomain, thereby presumably halting RNA chain elongation. As a result, this leads to inhibition of the SARS-CoV-2 RdRp activity. Nevertheless, our investigations employing pharmacophore modeling have revealed that butein necessitates modification, as it undergoes a loss of the chemical characteristics associated with hydrogen bond donor, aromatic hydrophobicity, and negative ionizability, as illustrated in Figure 2. The ionizable negative part plays a very important role in binding with magnesium ions that interact with the phosphate backbone of the diester and is part of the catalytic active site [64]. Butein lacks the necessary functional groups to compete effectively with remdesivir.

### 2.2. Antibacterial Activity of Erythrina

Table 2 summarizes the antibacterial activity of various Erythrina species. The methanol bark extract of *E. caffra* demonstrated remarkable efficacy against *S. aureus*, with a minimum inhibitory concentration (MIC) of 0.313 µg/mL [41]. In contrast, the ethanol bark extract of the same species showed only moderate inhibition at 39.1 µg/mL, indicating that the choice of extraction solvent significantly influences antibacterial potency [30]. Similarly, the methanol bark extract of *E. abyssinica* exhibited antibacterial activity with an MIC of 23 µg/mL. However, this effect was weaker compared to *E. caffra*, suggesting interspecies variations in phytochemical content of bioactive compounds [28].

Erybraedin, an isoflavonoid isolated from *E. lysistemon*, exhibited potent inhibitory activity against *S. aureus* (MIC: 2 µg/mL), underscoring the potential of Erythrina derivatives and isoflavonoids as antibacterial agents [35]. This finding is supported by the comparable activity of Eryvarin D from *E. fusca*, exhibiting comparable activity at 4 µg/mL against the same pathogen, suggesting consistency in antibacterial efficacy across species within the genus [27]. In contrast, Scandenone from *E. addisonae* showed weaker activity, with an MIC of 64 µg/mL. This highlights the variability in bioactivity depending on the compound class [32].

Ericristagallin, isolated from *Erythrina subumbrans*, exhibited remarkable activity against Methicillin-resistant *S. aureus* (MRSA) and Vancomycin-resistant *S. aureus* (VRSA), with minimum inhibitory concentrations (MICs) ranging between 0.39 and 1.56 µg/mL [65]. This inhibitory profile aligns with the findings from *Erythrina zeyheri*, where compounds, such as Erybraedin A and Eryzerin C, also exhibited potent activity against VRSA strains at MICs between 1.56 and 6.25 µg/mL, class-wide potency among prenylated isoflavonoids [36]. In contrast, the dichloromethane bark extract of *Erythrina stricta* exhibited diminished potency against MDRSA, with a minimum inhibitory concentration (MIC) of 31.25 µg/mL. This finding suggests a reduced efficacy of crude extracts in comparison to isolated compounds [23].

Furthermore, Erybraedin A from *E. lysistemon* exhibited consistent antibacterial activity against *S. epidermidis*, with a MIC of 2 µg/mL [35]. This is corroborated by the findings of reported comparable MIC values for Eryzerin C and Phaseollidin from the same species, suggesting these compounds contribute significantly to the antibacterial profile [32].

In contrast, within other bacterial groups, particularly Gram-negative pathogens, Neo-bavaisoflavone from *E. sigmoidea* exhibited inhibitory activity against *E. coli*, with minimum inhibitory concentrations (MICs) ranging from 8 to 32 µg/mL [25]. Similarly, flavanone derivatives from *Erythrina livingstoniana* exhibited minimum inhibitory concentrations (MICs) as low as 5 µg/mL [31]. In comparison, 6.8-diprenylgenistein and alpinumisoflavone from *E. caffra* with potent effects on *E. coli* and *K. pneumoniae*, showing MICs of 3.9–7.8 µg/mL [32]. These findings indicate consistent activity among prenylated flavonoids across Erythrina species against Enterobacteriaceae.

Lastly, Scandenone from *E. addisonae* demonstrated antibacterial activity against multiple strains, including *E. faecalis*, *B. subtilis*, and *E. cloacae*, with MIC values ranging from 16 to 64 µg/mL [8,32]. Although effective, these values indicate lower potency compared to compounds such as Erycristagallin or Erybraedin A. Collectively, these findings illustrate the broad-spectrum yet variable antibacterial potential of Erythrina, which is strongly influenced by the species differences, compound structure, and target pathogen.

### 2.3. Antifungal Activity of Erythrina

Table 3 summarizes the antifungal activity of various Erythrina species. A broad spectrum of activity has been documented, with particular efficacy against *C. albicans*, a common opportunistic fungal pathogen responsible for superficial infections, such as oral and vaginal candidiasis, and systemic infections in immunocompromised individuals. Among the tested extracts, the ethanol stem bark extract of *E. senegalensis* demonstrated the most potent inhibitory activity against *C. albicans*, with an MIC ranging between 4.00 and 15.63 µg/mL, indicating strong antifungal potential [42]. In contrast, other species such as *E. stricta* and *E. lysistemon* exhibited significantly higher MIC values (125 µg/mL and 25.000 µg/mL, respectively), highlighting the critical role of extraction method and plant part selection in modulating antifungal potency [23,37]. This variability in MIC underscores the influence of phytochemical diversity and compound abundance in different *Erythrina taxa*.

The antifungal spectrum of Erythrina extends beyond *C. albicans*, as several species have also exhibited inhibitory activity against other clinically relevant fungi, including *C. glabrata*, *C. krusei*, *Cryptococcus neoformans*, *Aspergillus niger*, *Penicillium camemberti*, *Scopulariopsis brevicaulis*, *Pyricularia oryzae*, and *Rhizopus stolonifer*. For example, *E. crista-galli* demonstrated activity against *C. krusei* with MIC values between 12.5 and 31.25 µg/mL, attributed to alkaloids such as erytharbine and erysotrine. Similarly, *E. sacleuxii* showed inhibition activity against *P. oryzae* through compounds such as prostratol C and orobol with a MIC of 20 µg/mL [31,44]. The identification of specific active constituents such as erypostyrene (from *E. poeppigiana*) and erysubin F (from *E. sacleuxii*) further reinforces the role of flavonoid and alkaloid subclasses within Erythrina as key contributors to its antifungal activity.

Collectively, the observed variation in antifungal efficacy, as evidenced by the wide MIC range, reflects the complexity of phytochemical interactions and highlights the need for further bioassay-guided fractionation to isolate and characterize specific bioactive compounds. These findings highlight the therapeutic potential of Erythrina as a valuable genus for the development of novel antifungal agents, particularly against resistant fungal pathogens.

### 2.4. Antiplasmodial Activity of Erythrina

Various Erythrina species have demonstrated activity against several Plasmodium species, including *Plasmodium falciparum*, the primary causative agent of malaria. Among them, the most potent compound reported is 5-hydroxysophoranone isolated from *E. stricta* and *E. subumbrans* with an IC_50_ of 2.5 µg/mL. The species also yielded other active compounds such as Erythrabbysin II (IC_50_ of 5.5 µg/mL), Soyaspongenol B (IC_50_; 4.6 µg/mL), and Erystagallin A (IC_50_; 3.8 µg/mL), which, although less potent than 5-hydroxysophoranone, still exhibited relatively low IC_50_ values [65]. In contrast, other Erythrina species demonstrated weaker antiplasmodial activity. For example, Lupeol isolated from *E. caffra* showed an IC_50_ of 41.7 µg/mL, while the aqueous stem bark extract of *E. abbysicina* had an IC_50_ of 47.74 µg/mL [49,78]. Beyond *P. falciparum*, Erythrina species have also shown activity against other Plasmodium species, as summarized in Table 4.

## 3. Discussion

Currently, effective treatments for infectious diseases are primarily based on well-established first-line therapies [84]. These include antiretroviral combinations for HIV, direct-acting antivirals for hepatitis, neuraminidase inhibitors for influenza, acyclovir derivatives for herpes simplex [85], β-lactams or glycopeptides for bacterial infections, azoles and polyenes for fungal diseases, and artemisinin-based combinations for malaria [86]. While these therapies have demonstrated efficacy, their utilization is frequently constrained by concerns related to resistance development, adverse effects, and accessibility. Consequently, there is an immediate need to investigate alternative sources, such as plant-derived compounds, which may present novel opportunities for complementary or replacement therapies.

**Figure 3:**
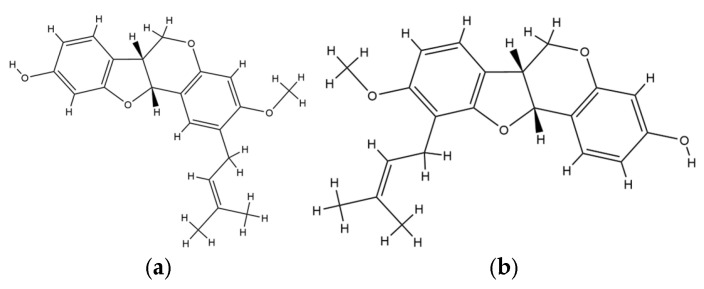
Chemical structure of (**a**) 3-O-methylcalopocarpin and (**b**) sandwicensin.

The current findings reaffirm that multiple Erythrina species possess remarkable antiviral properties across a broad viral spectrum, including HIV, SARS-CoV-2, hepatitis, influenza, herpes simplex virus, and dengue virus. Among these, *E. glauca* displayed the most potent anti-HIV activity, with 3-O-methylcalopocarpin exhibiting an EC_50_ of 0.2 µg/mL, followed by sandwicensin with 2 µg/mL [11]. This result indicates a high intrinsic antiviral capacity of *E. glauca* constituents, especially in disrupting HIV replication and entry pathways [87]. The implication of this observation lies in the compound’s capacity to attenuate the cytopathic effects exerted by HIV on human T-lymphocytes, suggesting a possible role in host cell protection [88]. Mechanistically, this activity may be linked to the flavonoid core structure, which is known to interfere with viral enzymatic activity, particularly reverse transcriptase inhibition, or disrupt viral protein assembly by binding at critical viral-host interaction domains. This high degree of activity aligns with previously reported studies on prenylated flavonoids as antiviral agents, further confirming the structural suitability of this class in targeting retroviruses. The specificity and low EC_50_ values highlight the potential of Erythrina species, especially *E. glauca*, as a promising phytochemical source for antiretroviral drug discovery [11]. The chemical structure of 3-O-methylcalopocarpin and sandwicensin is provided in Figure 3.

**Figure 4:**
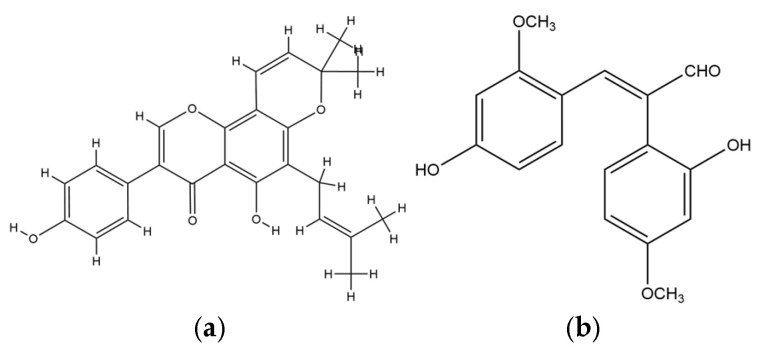
Chemical structure of (**a**) Osajin and (**b**) Erythradisson B.

A similarly strong antiviral potential is observed in *E. senegalensis*, particularly against hepatitis virus, where osajin demonstrated an EC_50_ of 67.54 µg/mL. Although less potent than anti-HIV candidates, its significance is magnified by its hepatoprotective effect via the inhibition of LDH leakage in CCl_4_-induced hepatotoxic models, pointing to a dual function of antiviral and organ-protective roles [89]. This duality suggests that osajin and related flavonoids may stabilize hepatocyte membranes or act as antioxidants, thereby indirectly mitigating virus-induced hepatic injury. Structurally, osajin contains prenyl side chains that may enhance its hepatic bioavailability and interaction with viral or host enzymes. These findings are consistent with literature reporting the cytoprotective action of prenylated isoflavones in models of oxidative stress and viral hepatitis, supporting its therapeutic relevance [20]. The chemical structure of osajin is provided in Figure 4a.

For the influenza virus, *E. addisonae* is the only species reported to exhibit activity with its compound erythradisson B, yielding a notably low EC_50_ of 8.8 µg/mL. The pharmacological implication is substantial, as this compound demonstrates a comparable inhibitory potential to early-generation neuraminidase inhibitors. The likely mechanism is direct inhibition of the neuraminidase enzyme, thereby preventing viral release and spread from infected epithelial cells [8]. Structurally, erythradisson B shares phenolic moieties that can engage the active site of neuraminidase via hydrogen bonding or hydrophobic interactions. This mirrors known inhibitory patterns of flavonoids in the sialidase enzyme pocket, consistent with earlier antiviral flavonoid research [90]. Chemical structure of erythradisson B is provided in Figure 4b.

The anti-herpes simplex virus activity of *Erythrina abbysinica* was particularly notable, with a stem bark aqueous extract achieving an IC_50_ of 27 µg/mL. Although not derived from a purified compound, the extract’s significant potency suggests the presence of bioactive alkaloids and coumarin-related constituents. The finding carries two key implications: first, it highlights the potential of crude plant materials in HSV inhibition; second, it emphasizes the need for compound-specific elucidation to validate the antiviral targets. Mechanistically, these constituents may inhibit HSV replication by disrupting viral DNA synthesis or glycoprotein-mediated entry, a mechanism commonly associated with erythrinan alkaloids. Compared to other plant-derived antivirals, the observed activity of *E. abbysinica* extract falls within a promising range for natural product leads [21].

**Figure 5:**
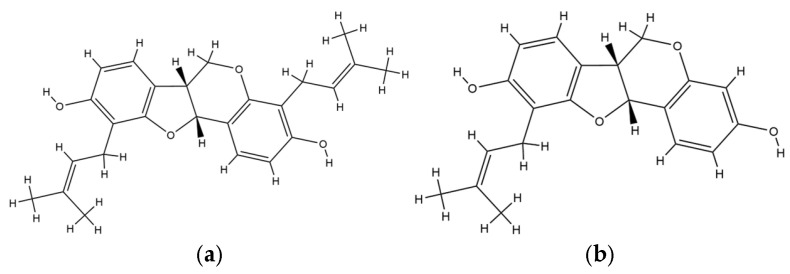
Chemical structure of (**a**) erybraedin A and (**b**) phaseollidin.

In the antibacterial domain, Erythrina species, particularly *E. caffra*, demonstrated highly potent activity, with a methanol bark extract achieving a MIC of 0.313 µg/mL against *S. aureus*. This potency indicates a pharmacological profile competitive with first-line antibiotics, underscoring its potential relevance in combating Gram-positive bacterial infections. The significance of these findings is amplified by the persistent global threat of methicillin-resistant *S. aureus* (MRSA), where treatment options are limited. The underlying mechanism is likely driven by membrane disruption and enzyme inhibition, especially targeting bacterial topoisomerases, as suggested by the presence of prenylated flavonoids such as erybraedin A and phaseollidin. These molecules, due to their lipophilic side chains, preferentially integrate into the lipid bilayer of Gram-positive bacteria, causing membrane destabilization and leakage. This observation aligns with published SAR (structure–activity relationship) studies, which indicate that prenylation significantly boosts flavonoid antibacterial efficacy [28]. The chemical structure of erybraedin A and phaseollidin is provided in Figure 5.

**Figure 6:**
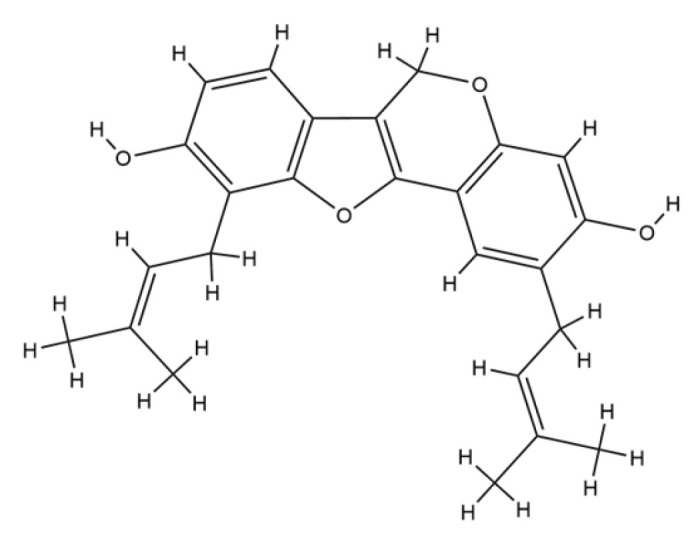
Chemical structure of ericristagallin.

Of particular significance is the consistent activity of Erythrina compounds against drug-resistant bacterial strains. For instance, ericristagallin from *E. subumbrans* exhibited minimum inhibitory concentrations (MICs) as low as 0.39 µg/mL against both MRSA and VRSA, suggesting its potential to circumvent resistance mechanisms. This activity suggests a non-classical mechanism, possibly bypassing the penicillin-binding proteins or efflux systems targeted by standard antibiotics [34]. The significance is further supported by the ability of *E. stricta* extracts to inhibit multidrug-resistant *S. aureus* at MICs of 31.25 µg/mL, reinforcing the hypothesis that these extracts contain multiple active constituents working synergistically. From a pharmacodynamic perspective, this multi-target approach is valuable as it reduces the evolutionary likelihood of resistance development, a major limitation of conventional monotherapeutic agents [36]. The structure of ericristagallin is provided in Figure 6.

**Figure 7:**
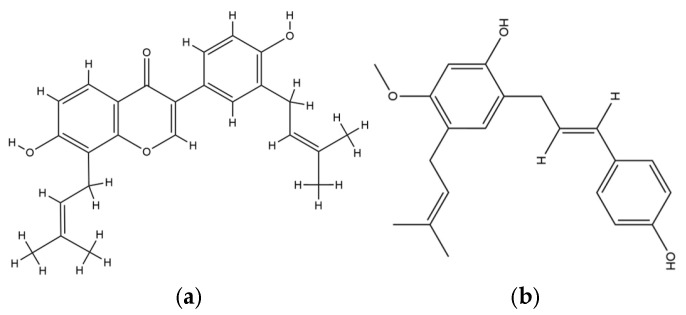
Chemical structure of (**a**) erysubin F and (**b**) erypostyrene.

In addition, the antifungal activity observed among various Erythrina species, notably *E. senegalensis*, underscores their clinical potential against fungal pathogens such as *C. albicans*, *C. glabrata*, and *Cryptococcus neoformans*. The MIC range of 4.00 to 15.63 µg/mL for *E. senegalensis* stem bark extract against *C. albicans* is within the threshold of therapeutic interest, particularly for resistant fungal strains. The pharmacological importance is elevated by the extract’s ability to disrupt biofilm formation, a known barrier to antifungal treatment. Mechanistically, bioactive compounds such as erypostyrene and erysubin F likely act through membrane destabilization or inhibition of ergosterol biosynthesis, impairing cell viability. The amphiphilic nature of these molecules facilitates their penetration into the fungal membrane, aligning with the literature on the antifungal role of prenylated pterocarpans [61,65]. The structure of erysubin F and erypostyrene is provided in Figure 7.

**Figure 8:**
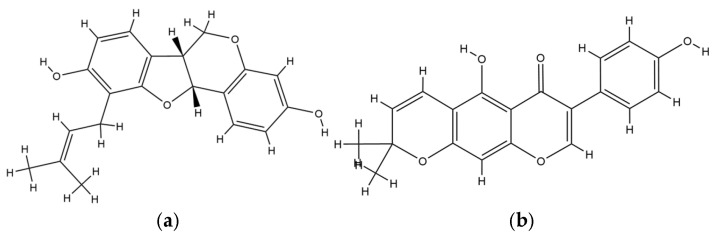
Chemical structure of (**a**) phaseollidin and (**b**) alpinumisoflavone.

Finally, the antiplasmodial activity of Erythrina species provides further evidence of the genus’s broad-spectrum antimicrobial capacity. Notably, *E. sacleuxii* methanolic root bark extract exhibited an exceptionally low IC_50_ of 0.45 µg/mL against *Plasmodium falciparum*, and pure compounds such as phaseollidin and alpinumisoflavone demonstrated IC_50_ values below 2 µg/mL [69]. This indicates a high level of cytotoxic efficacy toward the parasite at low concentrations, comparable to established antimalarials. The likely mechanisms include interference with hemozoin formation, mitochondrial dysfunction, or oxidative damage induction [91]. The presence of multiple aromatic rings and phenolic hydroxyl groups in these compounds enhances their interaction with parasite biomolecules, particularly via redox cycling and mitochondrial disruption, as supported by analogous flavonoid-based antimalarial studies [92,93]. The structure of phaseollidin and alpinumisoflavone is provided in Figure 8.

Together, these findings affirm the pharmacological potential of Erythrina species as a reservoir of antiviral, antibacterial, antifungal, and antiplasmodial agents. The spectrum of activity observed across different pathogens is not only a testament to the genus’s chemical richness but also provides a strong rationale for further isolation, characterization, and preclinical evaluation of its constituents in the development of novel therapies.

## 4. Methodology

**Figure 9:**
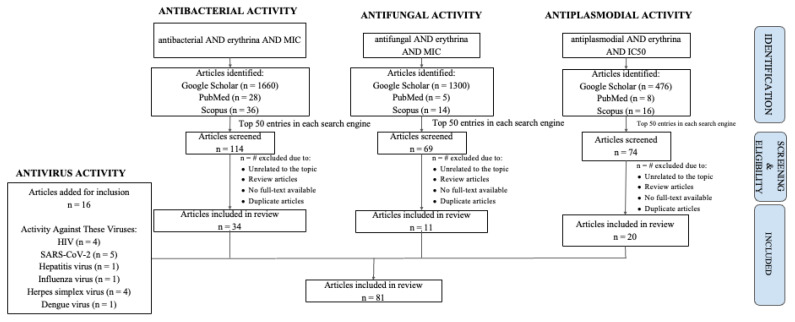
Flow chart of methodology.

This review adopts a narrative-critical approach to comprehensively evaluate the antiviral and antimicrobial potential of Erythrina species, emphasizing phytochemical diversity and pharmacological relevance. A structured literature search was conducted across PubMed, Scopus, and Google Scholar up to June 2025 using targeted Boolean combinations, including “Erythrina AND antiviral,” “Erythrina AND antibacterial AND MIC,” “Erythrina AND antifungal AND MIC,” and “Erythrina AND antiplasmodial AND IC_50_,” alongside virus-specific queries (e.g., “HIV”, “SARS-CoV-2”). Titles, abstracts, and full texts were screened, with the top 50 entries per keyword set reviewed for eligibility. Peer-reviewed studies reporting quantitative in vitro or in vivo data on extracts or isolated compounds from Erythrina—with EC_50._ IC_50._ or MIC values were included. Non-pharmacological, non-English, or insufficiently detailed studies were excluded. Relevant data were extracted; categorized by pathogen type, species, and compound; and synthesized to identify mechanistic trends and comparative efficacy. The literature selection process is visually summarized in Figure 9, which provides a transparent overview of article identification, screening, and inclusion. While not adhering to PRISMA due to the narrative scope, the methodology reflects academic rigor, analytical coherence, and thematic precision in evaluating the phytotherapeutic promise of Erythrina in infectious disease contexts.

## 5. Conclusions

This comprehensive review systematically compiles and analyzes data across a wide spectrum of infectious targets to elucidate the antiviral, antibacterial, antifungal, and antiplasmodial potentials of the Erythrina genus. The literature demonstrates that multiple Erythrina species exhibit remarkable and often species-specific activities against clinically significant pathogens, including HIV, SARS-CoV-2, *S. aureus*, *C. albicans*, and *P. falcipa-rum*. These effects are primarily attributed to structurally diverse phytoconstituents, such as prenylated flavonoids, pterocarpans, and erythrina alkaloids, which act through mechanisms including enzymatic inhibition, membrane disruption, and modulation of microbial virulence. Notably, the genus consistently displays inhibitory profiles against multidrug-resistant bacteria and biofilm-forming fungi, positioning Erythrina-derived compounds as promising candidates for next-generation anti-infective development.

## Figures and Tables

**Table 1:** Antiviral activity of Erythrina.

No	Species	Compounds/Extracts	Virus	Ref.
1	*E*. *glauca*	Sandwicensin (EC_50_: 2 µg/mL) 3-O-methylcalopocarpin (EC_50_: 0.2 µg/mL)	HIV	[11]
2	*E*. *lysistemon*	5-deoxyglyasperin F (EC_50_: 11.5 µg/mL) 2′-hydroxyneobavaisoflavanone (EC_50_: 7.6 µg/mL)	HIV	[11]
3	*E*. *abbysinica*	Crude alkaloid fraction (EC_50_: 53 µg/mL). The compounds identified were erythraline, erysodine, erysotrine, 8-oxoerythraline, and 11-methoxyerysodine	HIV	[12]
4	*E*. *variegata*	Apigetrin (EC_50_: 100.59 µg/mL)	HIV	[17]
5	*E*. *senegalensis*	Auriculatin (EC_50_: 1.47 µg/mL) Erysenegalensein O (EC_50_: 2.19 µg/mL) Erysenegalensein D (EC_50_: 1.10 µg/mL) Erysenegalensein N (EC_50_: 1.97 µg/mL) 6.8-diprenylgenistein (EC_50_: 0.203 µg/mL)	HIV	[10]
6	*E*. *subumbrans*	Gangetinin	SARS-CoV-2	[14]
7	*E. sigmoidea*	Gangetin	SARS-CoV-2	[13]
8	*E. senagelensis*	Erybraedin D	SARS-CoV-2	[16]
9	*E. variegata*	Orientanol E	SARS-CoV-2	[62]
10	*E. caffra*	Erycaffra F	SARS-CoV-2	[18]
11	*E. speciosa*	Vitexin (EC_50_: 125 µg/mL)	Hepatitis Virus	[19]
12	*E. senegalensis*	2. 3-dihydro-2′-hydroxyosajin (EC_50_: 71.8 ± 1.45 µg/mL) osajin (67.54 ± 3.56 µg/mL) 6.8-diprenylgenistein (69.41 ± 2.56 µg/mL)	Hepatitis Virus	[20]
13	*E. addisonae*	Erythradisson B (EC_50_: 8.8 µg/mL) Licoagrochalcone (EC_50_: 21.51 µg/mL) Abyssinone VI (EC_50_: 26.44 µg/mL) 5-prenylbutein (EC_50_: 21.93 µg/mL)	Influenza	[8]
14	*E. abbysinica*	Stem bark aqueous extract (IC_50_: 27 µg/mL)	HSV	[21]
15	*E. speciosa*	Vitexin (EC_50_: 64 µg/mL)	HSV	[19]
16	*E. fusca*	Stem bark decoction (EC_50_: 243 ± 10.9 µg/mL)	HSV	[15]
17	*E. poeppigiana*	Stem bark decoction (EC_50_: 147.6 ± 4.3 µg/mL)	HSV	[22]
18	*E. variegata*	Ericristagallin, Osajin, Sigmoidin A, Sigmoidin B, Sigmoidin C, Robustone, Abyssinone, Eryvarin A, Eryvarin B, Euchrenone, Lupiwighteone, Laburnetine	Dengue Virus	[61]

**Table 2:** Antibacterial activity of Erythrina.

Bacteria	*Erythrina*	Compounds	MIC (µg/mL)	Ref
Staphylococcus				
*S. aureus*	*E. caffra*	6.8-diprenylgenistein	7.8	[24]
		Ethanol bark extract (specific compounds are unknown)	39.1	[30]
		Methanol bark extract (specific compounds are unknown)	0.313	[41]
	*E. lysistemon*	Erybraedin A	2	[35]
		Phaseollidin	10	
		Abyssinone V-4′-methyl-ether	59	
		Eryzerin C	5	
		Alpumisoflavone	31	
		Lysisteisoflavone	62	
	*E. senegalensis*	Ethanol leaf extract (specific compounds are unknown)	25	[35]
	*E. stricta*	Dichloromethane bark extract (specific compounds are unknown)	7.81	[23]
	*E. fusca*	Sandwicensin	8	[27]
		Erythrabbysin A	64	
		Erythrabbysin I	64	
		Eryvarin D	4	
		Scandeone	8	
	*E. abyssinica*	Methanol bark extract (specific compounds are unknown)	23	[28]
		Ethanol bark extract (specific compounds are unknown)	62.5	[38]
		Ethyl acetate bark extract (specific compounds are unknown)	83.3	
	*E. amazonia*	Methanol bark extract (specific compounds are unknown)	75	[33]
	*E. addisonae*	Scandenone	64	[32]
Methicillin-Resistant *S. aureus* (MRSA)	*E. subumbrans*	Ericristagallin	0.39–1.56	[65]
	*E. poeppigiana*	Eryvarin D	12.5	[40]
		3.9-dihydroxy-10-γ,γ- dimethylallyl- 6a,11adehydropterocarpan	12.5	
		Isolupalpigenin	1.56–3.13	[66]
		Erythrinin B	6.25	
		Erypostyrene	6.25	[37]
		Sandwicensin	6.25–12.5	
		Erypoegin A	25	
		Dimethylmedicarpin	50	
		Angolensin	50	
	*E. sacleuxii*	Erysubin F	15.4	[29]
		7.4′-dihydroxy-8.3′-diprenylflavone	20.5	
	*E. variegata*	Eryvrain V	12.5–25	[39]
		Eryvarin W	1.56–3.13	
		Eryvarin X	0.78–1.56	
		Bidwillon B	3.13–6.25	[67]
		Eryvarin Q	3.13–6.25	[68]
	*E. stricta*	Dichloromethane bark extract (specific compounds are unknown)	31.25	[23]
	*E. herbacea*	Erybacin A	50	[15]
		Erybacin B	12.5	
		Eryvariestyrene	12.5	
		Glyasperin F	50	
		Bidwillol A	12.5	
		Phaseollinisoflavan	50	
		Erythbidin	50	
		Phaseollidin isoflavan	12.5	
		Eryvarin L	25	
		Glabrocoumarone A	12.5	
	*E. fusca*	Sandwicensin	16	[27]
		Erythrabbysin A	32	
		Erythrabbysin I	64	
		Eryvarin D	4	
Vancomycin-Resistant *S. aureus* (VRSA)	*E. subumbrans*	Erycristagallin	0.39–1.56	[65]
	*E. zeyheri*	Erybraedin A	1.56–3.13	[36]
		Eryzerin A	12.5–25	
		Eryzerin C	6.25	
		Eryzerin D	12.5	
		Eryzerin E	12.5	
Multidrug-Resistant *S. aureus* (MDRSA)	*E. stricta*	Dichloromethane bark extract (specific compounds are unknown)	31.25	[23]
*S. epidermidis*	*E. lysistemon*	Dichloromethane bark extract (specific compounds are unknown)	40	[69]
		Erybraedin A	2	[35]
		Phaseollidin	5	
		Eryzerin C	2	
		Lysisteisoflavone	26	
	*E. addisonae*	Scandenone	32	[32]
Actinomyces				
*A. viscosus*	*E. variegata*	2-(γ,γ-dimethylallyl)-6a-hydroxyphaseollidin	3.13	[70]
		Erystagallin A	3.13	
		Erycristagallin	1.56	
Micrococcus				
*M. luteus*	*E. caffra*	Ethanol bark extract (specific compounds are unknown)	39.1	[30]
		Methanol bark extract (specific compounds are unknown)	0.156	[41]
Enterococcus				
*E. faecalis*	*E. caffra*	Ethanol bark extract (specific compounds are unknown)	39.1	[30]
		Methanol bark extract (specific compounds are unknown)	0.156	[41]
		Acetone leaf extract (specific compounds are unknown)	80	[71]
	*E. addisonae*	Scandenone	64	[32]
Bacillus				
*B. cereus*	*E. lysistemon*	Erybraedin A	1	[35]
		Phaseollidin	10	
		Abyssinone V-4′-methyl-ether	26	
		Eryzerin C	10	
		Alpumisoflavone	31	
		Lysisteisoflavone	2	
	*E. caffra*	Methanol bark extract (specific compounds are unknown)	78	[41]
	*E. addisonae*	Scandenone	16	[32]
*B. pumilus*	*E. caffra*	Methanol bark extract (specific compounds are unknown)	39.1	[41]
*B. brevis*	*E. cristagalli*	Coumestrol	4.4	[72]
		Genistein	13.5	
		Daidzein	35	
*B subtilis*	*E. addisonae*	Scandenone	16	[32]
Mycobacterium				
*M. tuberculosis*	*E. stricta*	Erythrabbysin II	50	[65]
		Erystagallin A	12.5	
		Erythrabbysin-1	50	
		5-hydroxysophoranone	12.5	
		Sandwicensin	50	
	*E. subumbrans*	Erybraedin A	25	
		Erythrabbysin II	50	
		Erystagallin A	12.5	
		Erythrabbysin-1	50	
		Erycristagallin	12.5	
		5-hydroxysophoranone	12.5	
		Erysubin F	12.5	
Rifampicin-Resistant *M. tuberculosis*	*E. abyssinica*	Methanol bark extract (specific compounds are unknown)	390	[73]
*M. smegmatis*	*E. addisonae*	Scandenone	100	[32]
Propionibacterium				
*P. acnes*	*E. lysistemon*	Methanol: Dichloromethane (1:1) leaf extract (specific compounds are unknown)	80	[74]
Escherichia				
*E. coli*	*E. sigmoidea*	Neobavaisoflavone	8–32	[25]
	*E. livingstoniana*	5.7.3′-trihydroxy-4′-methoxy-5′-(3-methylbut2-enyl)flavanone	5	[75]
		7.3′-dihydroxy-4′-methoxy-5′-(3- methylbut-2-enyl)flavanone	5	
		(3S,3″R)-7-hydroxy-2′-methoxy-[3″-hydroxy-2″,2″-dimethylpyrano (3′,4′)] isoflavan	63	[76]
	*E. caffra*	Abyssione-V 4′-O-methyl ether	3.9	[24]
		6.8-diprenylgenistein	7.8	
		Alpinumisoflavone	3.9	
		Methanol bark extract (specific compounds are unknown)	0.02	[41]
	*E. lysistemon*	Erybraedin A	2	[35]
		Phaseollidin	20	
		Eryzerin C	5	
		Lysisteisoflavone	6	
	*E. senegalensis*	Ethanol leaf extract (specific compounds are unknown)	25	[26]
	*E. addisonae*	Scandenone	64	[32]
Enterobacter				
*E. cloacae*	*E. sigmoidea*	Neoisoflavone	8	[25]
*E. aerogenes*	*E. addisonae*	Scandenone	64	[32]
*E. cloaca*	*E. addisonae*	Scandenone	64	
Klebsiella				
*K. pneumoniae*	*E. sigmoidea*	Neoisoflavone	8	[25]
	*E. caffra*	Abyssione-V 4′-O-methyl ether	3.9	[24]
		6.8-diprenylgenistein	7.8	
		Alpinumisofl avone	3.9	
	*E. senegalensis*	Ethanol leaf extract (specific compounds are unknown)	6.25	[26]
Pseudomonas				
*P. aeruginosa*	*E. sigmoidea*	Neoisoflavone	8	[25]
	*E. lysistemon*	Erybraedin A	20	[35]
		Phaseollidin	20	
		Cristacarpin	78	
		Eryzerin C	5	
		Alpumisoflavone	20	
		Lysisteisoflavone	31	
Proteus				
*P. vulgaris*	*E. caffra*	Ethanol bark extract	156.3	[30]
Salmonella				
*S. typhi*	*E. caffra*	Methanol bark extract (specific compounds are unknown)	0.02	[41]
*S. enteretidis*	*E. senegalensis*	Calopocarpin	62.5	[77]
	*E. abyssinica*	Methanol bark extract (specific compounds are unknown)	29	[28]

**Table 3:** Antifungal activity of Erythrina.

Fungi	*Erythrina* Species	Compounds	MIC (µg/mL)	Ref
*C. albicans*	*E. indica*	Ethanol leaf extract (specific compounds are unknown)	62.5	[43]
	*E. senegalensis*	Ethanol stem bark extract (specific compounds are unknown)	4.00–15.63	[42]
	*E. stricta*	Dichloromethane bark extract (specific compounds are unknown)	125	[23]
	*E. poeppigiana*	Erypostyrene	50	[37]
	*E. sacleuxii*	Erysubin F	>32.021	[29]
	*E. lysistemon*	Aqueous extracts (specific compounds are unknown)	25,000	[45]
*C. glabrata*	*E. senegalensis*	Ethanol stem bark extract (specific compounds are unknown)	3.91–62.5	[42]
*C. krusei*	*E. crista-galli*	Erytharbine, erysotrine, erythratidine N-oxide	12.5–31.25	[44]
*C. neoformans*	*E. senegalensis*	Hydroalcoholic leaves extract (specific compounds are unknown)	3120	[46]
*A. niger*	*E. senegalensis*	Lectin seeds extract (specific compounds are unknown	400	[5]
	*E. lanceolata*	Dichloromethane bark extract (specific compounds are unknown)	1250	[31]
*P. camembreti*	*E. senegalensis*	Lectin seeds extract (specific compounds are unknown	200	[5]
*S. brevicaulis*	*E. senegalensis*	Lectin seeds extract (specific compounds are unknown	200	[5]
*P. oryzae*	*E. sacleuxii*	Prostratol C and orobol	20	[31]
*R. stolonifer*	*E. lanceolata*	Dichloromethane bark extract (specific compounds are unknown)	625	[47]

**Table 4:** Antiplasmodial activity of Erythrina.

Plasmodium	*Erythrina* Species	Compounds	IC_50_ (µg/mL)	Ref
*P. falciparum*	*E. stricta*	Erythrabbysin II	5.5	[65]
		Erystagallin A	3.8	
		5-hydroxysophoranone	2.5	
		Soyaspongenol B	4.6	
	*E. subumbrans*	Erybraedin A	3.4	
		Erythrabbysin II	5.5	
		Erystagallin A	3.8	
		5-hydroxysophoranone	2.5	
		Erysubin F	3.2	
		Soyaspongenol B	4.6	
	*E. latissima*	Erysodine	6.5 ± 4.7	[50]
		Erysovine	4.1 ± 0.6	
		Erysotrine	20.6 ± 8.6	
		Erythraline	7.3 ± 4.9	
	*E. sacleuxii*	Methanol stem bark extract (specific compounds are unknown)	1.78 ± 0.93	[48]
		Methanol leaf extract (specific compounds are unknown)	24.59 ± 10.54	
		Methanol root bark extract (specific compounds are unknown)	0.45 ± 0.09	
		Acetone stem bark extract (specific compounds are unknown)	3.8 ± 0.9	[51]
		Acetone root bark extract (specific compounds are unknown)	2.2 ± 0.6	
	*E. abbysinica*	Methanol stem bark extract (specific compounds are unknown)	37.37 ± 6.46	[49]
		Dichloromethane stem bark extract (specific compounds are unknown)	5.37 ± 1.59	
		aqueous stem bark extract (specific compounds are unknown)	47.74 ± 9.15	
	*E. caffra*	*Erythrina*sinate B	24.4 ± 2.63	[78]
		Lupeol	41.7 ± 9.74	
	*E. ovalifolia*	Erythrisenegalone	1.69	[52]
		Alpinumisoflavone	1.98	
		Phaseollidin	1.66	
		Sandwicensin	1.83	
	*E. burttii*	Buttinol A	7.6 ± 0.3	[56]
		Buttinol B	19.1 ± 0.6	
		Buttinol C	9.3 ± 0.9	
		Buttinol H	13.3 ± 2.5	
		Buttinol D	4.9 ± 0.3	
		4-O-Methylsigmoidin B	12.4 ± 1.7	
		Abbysinone V	5.7 ± 0.5	
		Abbysinone V methyl ether	10.7 ± 2.4	
		Calocarpin	19.4 ± 1.8	
	*E. senegalensis*	Ethanol stem bark extract (specific compounds are unknown)	1.82	[54]
	*E. fusca*	Methanol stem bark extract (specific compounds are unknown)	13	[55]
		N-hexane stem bark extract (specific compounds are unknown)	21	
		Chloroform stem bark extract (specific compounds are unknown)	22	
		Aqueous stem bark extract (specific compounds are unknown)	5	
	*E. sigmoidea*	Ethanol stem bark extract (specific compounds are unknown)	6.44 ± 0.08	[53]
		Aqueous stem bark extract (specific compounds are unknown)	29.51 ± 3.63	
	*E. haerdii*	Ethanol leaf extract (specific compounds are unknown)	25.6 ± 2.5	[58]
		Ethanol root bark extract (specific compounds are unknown)	11.0 ± 0.7	
		Ethanol stem bark extract (specific compounds are unknown)	8.6 ± 0.8	
	*E. variegata*	Ethyl acetate leaf extract (specific compounds are unknown)	16.7	[59]
		N-butanol leaf extract (specific compounds are unknown)	13.2	
		10.11-dioxoerythratidine	9.3	
		terpenoid pentacyclic glycoside	1.8	
	*E. corallodendron*	Isoschaftoside	1.702	[57]
		Vicenin II	1.744	
Chloroquine-Resistant *P. falciparum*	*E. abbysinica*	Methanol stem bark extract (specific compounds are unknown)	34.13 ± 9.79	[49]
		Dichloromethane stem bark extract (specific compounds are unknown)	6.99 ± 0.76	
		Aqueous stem bark extract (specific compounds are unknown)	50.11 ± 10.23	
	*E. schliebenii*	Ethanol root extract (specific compounds are unknown)	1.87 ± 0.44	[79]
		Aqueous stem bark extract (specific compounds are unknown)	7.04 ± 0.72	
	*E. burttii*	Buttinol A	8.5 ± 0.6	[56]
		Buttinol B	21.1 ± 0.8	
		Buttinol C	9.1 ± 1.2	
		Buttinol H	20.3 ± 4.1	
		Buttinol D	6.1 ± 1.5	
		4-O-Methylsigmoidin B	12.7 ± 2.3	
		Abbysinone V	6.6 ± 1.3	
		Abbysinone V methyl ether	11.9 ± 2.1	
		Calocarpin	17 ± 1.5	
	*E. lysistemon*	aqueous stem bark extract (specific compounds are unknown)	8.27	[80]
	*E. fusca*	Methanol stem bark extract (specific compounds are unknown)	8	[55]
		N-hexane stem bark extract (specific compounds are unknown)	5	
		Chloroform stem bark extract (specific compounds are unknown)	13	
		Aqueous stem bark extract (specific compounds are unknown)	18	
	*E. sacleuxii*	Acetone stem bark extract (specific compounds are unknown)	6.3 ± 1.4	[51]
		Acetone root bark extract (specific compounds are unknown)	1.34 ± 0.3	
	*E. haerdii*	Ethanol leaf extract (specific compounds are unknown)	36.4 ± 1.9	[58]
		Ethanol root bark extract (specific compounds are unknown)	15.2 ± 0.9	
		Ethanol stem bark extract (specific compounds are unknown)	7.6 ± 0.7	
	*E. variegata*	Methanol leaf extract (specific compounds are unknown)	6.8	[59]
		Ethyl acetate leaf extract (specific compounds are unknown)	26.5	
		N-butanol leaf extract (specific compounds are unknown)	5.1	
		10.11-dioxoerythratidine	3.3	
		terpenoid pentacyclic glycoside	3.2	
	*E. corallodendron*	Isoschaftoside	1.1049	[57]
		Vicenin II	1.4099	
Multidrug-Resistant *P. falciparum*	*E. senegalensis*	Methanol stem bark extract (specific compounds are unknown)	99.6 ± 1.25	[81]
	*E. abbysinica*	Methanol stem bark extract (specific compounds are unknown)	7.81	[82]
	*E. subumbrans*	Vogelin C	2.8	[34]
		Lespedezaflavanone B	3.7	
		Abbysinone V	7.0	
	*E. sigmoidea*	Ethanol stem bark extract (specific compounds are unknown)	7.53 ± 0.22	[53]
		Aqueous stem bark extract (specific compounds are unknown)	35.23 ± 3.17	
	*E. fusca*	Lonchocarpol A	9.18	[83]
		Phaseollidin	9.09	

## Data Availability

No new data were created or analyzed in this study. Data sharing is not applicable to this article.
